# *Echinococcus* spp. and genotypes infecting humans in Tibet Autonomous Region of China: a molecular investigation with near-complete/complete mitochondrial sequences

**DOI:** 10.1186/s13071-022-05199-6

**Published:** 2022-03-05

**Authors:** Yanping Zhao, Dunzhu Gesang, Li Wan, Jiandong Li, Gezhen Qiangba, Wangmu Danzeng, Zhuoga Basang, Nibu Renzhen, Jiefang Yin, Quzhen Gongsang, Huimin Cai, Huasheng Pang, Daxi Wang, Asan, Qingda Zhang, Junhua Li, Weijun Chen

**Affiliations:** 1 BGI-Shenzhen, Shenzhen, 518083 China; 2 Shenzhen Key Laboratory of Unknown Pathogen Identification, BGI-Shenzhen, Shenzhen, 518083 China; 3 NHC Key Laboratory of Echinococcosis Prevention and Control, Lhasa, 850010 China; 4 Second People’s Hospital of Tibet Autonomous Region, Lhasa, 850000 China; 5 College of Life Sciences, University of Chinese Academy of Sciences, Shenzhen, 518083 China; 6 BGI-Tibet, BGI-Shenzhen, Lhasa, 850000 China; 7 Tibet Centre for Disease Control and Prevention, Lhasa, 850010 China; 8 BGI PathoGenesis Pharmaceutical Technology, BGI-Shenzhen, Shenzhen, 518083 China

**Keywords:** Cystic echinococcosis, Alveolar echinococcosis, Tibet Autonomous Region, *Echinococcus granulosus*, *Echinococcus multilocularis*, *Echinococcus canadensis*, Mitochondrial genome, Next-generation sequencing

## Abstract

**Background:**

Molecular markers are essential to identify *Echinococcus* species and genotypes in areas with multiple *Echinococcus* species to understand their epidemiology and pathology. Tibet Autonomous Region (TAR) is one of the areas worst hit by echinococcosis. However, molecular epidemiology is still missing among echinococcosis patients in TAR. This research explored the *Echinococcus* species and genotypes infecting humans in TAR and the population diversity and the possible origin of G1 in TAR.

**Methods:**

Cyst samples were collected in one echinococcosis-designated hospital in TAR. *Echinococcus* species and genotypes were identified through a maximum-likelihood approach with near-complete/complete mtDNA using IQ-TREE. Phylogenetic networks were built with PopART, and the phylogeographical diffusion pattern was identified using a Bayesian discrete phylogeographic method.

**Results:**

Using phylogenetic trees made with near-complete/complete mtDNA obtained from 92 cysts from TAR patients, the *Echinococcus* species and genotypes infecting humans in TAR were identified as *Echinococcus granulosus* (s.s.) G1 (81, 88.04%), accounting for the majority, followed by G6 of the *E. canadensis* cluster (6, 6.52%), *E. granulosus* (s.s.) G3 (3, 3.26%), and *E. multilocularis* (2, 2.17%). An expansion trend and a possible recent bottleneck event were confirmed among the G1 samples in TAR. Adding the other near-complete mtDNA of G1 samples globally from the literature, we identified the possible phylogeographic origin of the G1 samples in TAR as Turkey.

**Conclusions:**

Using near-complete/complete mtDNA sequences of *Echinococcus* spp. obtained from echinococcosis patients, a variety of *Echinococcus* species and genotypes infecting humans throughout TAR were identified. As far as we know, this is the first comprehensive molecular investigation of *Echinococcus* species and genotypes infecting humans throughout TAR. We identified, for the first time to our knowledge, the possible origin of the G1 in TAR. We also enriched the long mtDNA database of *Echinococcus* spp. and added two complete *E. multilocularis* mtDNA sequences from human patients. These findings will improve our knowledge of echinococcosis, help to refine the targeted echinococcosis control measures, and serve as a valuable baseline for monitoring the *Echinococcus* species and genotypes mutations and trends of the *Echinococcus* spp. population in TAR.

**Graphical Abstract:**

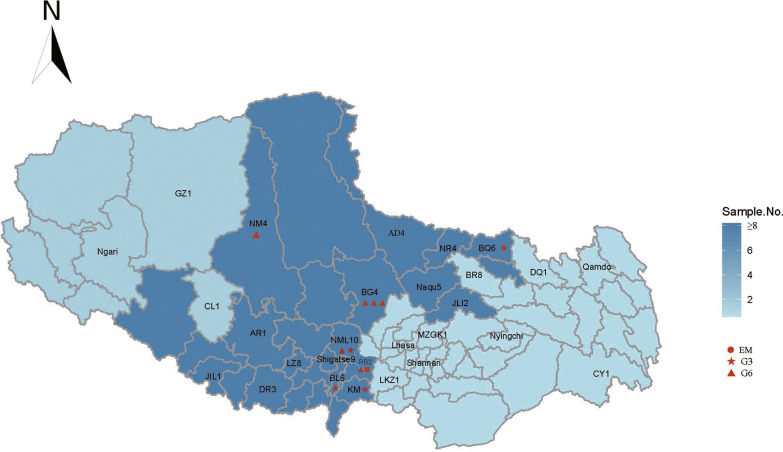

**Supplementary Information:**

The online version contains supplementary material available at 10.1186/s13071-022-05199-6.

## Background

Echinococcosis is 1 of the 17 neglected tropical diseases prioritized to be controlled by the World Health Organization (WHO) [1]. Two main echinococcoses affect humans, namely cystic echinococcosis (CE), caused by *Echinococcus granulosus* (s.l.), and alveolar echinococcosis (AE), caused by *Echinococcus multilocularis* (*E. multilocularis*) [2]. These parasites rank second and third in the global food-borne parasite list of the Food and Agriculture Organization (FAO) [3].

As the treatment and prevention vary between AE and CE, accurate identification between them is very important. Even within CE, which was thought to be due to mostly *Echinococcus granulosus* (s.s.), it was later found that 11.07% of CE cases were due to *E. canadensis* [4, 5]. A study in Mongolia identified all 18 children were infected by *E. canadensis* (94.4% G6/G7 of the *E. canadensis* cluster) [6]. Without molecular identification, our knowledge of CE was derived from a mixture of *Echinococcus* species and genotypes. Molecular markers are essential to detect cases in areas with multiple *Echinococcus* species and genotypes to understand their epidemiology, pathology, and infectivity to humans to establish corresponding control strategies [4, 7, 8]. Using short fragments, mainly through PCR, molecular epidemiology has been applied to identify the main circulating *Echinococcus* spp. infecting humans [5, 9–11]. However, short fragments of mitochondrial DNA (mtDNA) often could not distinguish closely related genotypes including G1/G3 and G6/G7 of the *E. canadensis* cluster [12, 13]. These genotypes were classified based on mtDNA and named following the hosts identified. G1 was known as the sheep strain and was found worldwide, but it was reported not only from sheep, but also humans, kangaroos, dingo, cattle camel, pigs, and goats [14]. G3 was known as the buffalo strain and was first found in India [14]. Later, G3 was identified as the main genotype for human infection in Pakistan and North India [11, 15]. G6 was known as the camel strain and was identified from camels in Somalia and Sudan, goats in Turkana, Kenya, and cattle and humans in China [14, 16, 17]. G7 was known as the pig strain and was first reported in Poland but was also reported from pigs in Bolivia and humans in Heilongjiang, China [14, 18, 19]. These strains/genotypes were mainly classified by sequencing partial mtDNA [20].

Western China is heavily affected by echinococcosis, accounting for 40% CE DALYs and 91% of the annual AE incidence globally [21–23]. Of the affected provinces, Tibet Autonomous Region (TAR) ranked top in echinococcosis prevalence [24]. Although there was a prevalence study in TAR using ultrasound diagnostics, molecular markers were seldom used to identify the *Echinococcus* species and genotypes infecting the echinococcosis patients in TAR. Previous molecular epidemiological studies in this region often included animal samples with very few samples from humans; even when samples from patients were involved, they were mainly patients from Qinghai or Sichuan Provinces, but rarely patients from TAR; besides, previous studies were using short fragments of the mtDNA such as *cox*1, a fragment of *cox*1, *nad*1, and *atp*6 [5, 9, 25–27], which could not confidently allocate the samples into specific genotypes and could only generate networks with lower resolution; therefore, the exact *Echinococcus* species and genotypes infecting humans in TAR remain unknown. The accurate allocation of the samples into different species and genotypes is the prerequisite for the exploration of the epidemiology, pathology, and infectivity to humans of the different species and genotypes. After the exact species and genotypes infecting humans are identified, it is possible to explore the association between the genotypes and ultrasound presentation. The accumulated knowledge would lead to more accurate diagnosis and control measures [8]. In this study, we identified the *Echinococcus* species and genotypes infecting humans in TAR through near-complete/complete mitochondrial sequences and explored the corresponding ultrasound presentation and the genetic variability as well as the possible origin of the most common *Echinococcus* genotype in TAR.

## Methods

### Study area

TAR is a western province with seven prefectures and 3 million people; its average altitude is > 4000 m [28]. TAR borders Xinjiang Uygur Autonomous Region to the north, Qinghai and Sichuan Provinces to the east, and Yunnan Province to the south (only a short section).

### Sample collection

Patients who underwent surgery to remove hydatid cysts in the Second People’s Hospital of TAR from 2018 to 2019 were invited to participate. After surgery, the cysts were checked for the presence of protoscoleces under a microscope and stored in 95% ethanol. Patient demographic information and ultrasound classification of the cyst were recorded and analyzed with R version 4.1.1. Maps were also drawn with R version 4.1.1. CE and AE differential diagnoses were made with ultrasound following the guidelines [29, 30]. Species identification was carried out with qPCR, which was based on the primers designed by Boufana et al., which could identify three kinds of *Echinococcus* species, namely *E. granulosus* (G1), *E. multilocularis*, and *E. shiquicus* [31]. For each sample, three replicates were made, and the mean of the three was used as the final result.

### DNA extraction and sequencing

Genomic DNA was extracted from protoscoleces (in fertile cysts) and/or germinal layers (in infertile cysts) from cysts taken from echinococcosis patients using the phenol-chloroform method [32]. The extracted DNA was used for sequencing on BGISEQ-500/DIPSEQ-T1 platforms (MGI, Shenzhen, China) if the DNA amount was ≥ 0.7 µg [33]. Different from the Sanger method, BGISEQ-500/DIPSEQ-T1 sequencing platform uses DNA nanoball sequencing technology and uses Rolling circle replication to amplify DNA linearly. The sequence information was later obtained through sequencing-by-synthesis technology. The minimum DNA amount for samples to be handled by BGISEQ-500/DIPSEQ-T1 was 0.7 µg; thus, if the DNA amount was below this value, we used the PCR method first to increase the concentration.

### PCR amplification and mitochondrial genome sequencing

For the rest of the samples with DNA < 0.7 µg, 13 published primer pairs were used to amplify mitochondrial genes through PCR, and the amplification products were sequenced [12]. As most of the PCR primers were designed for G1/G3 and tested with G6/G7 of the *E. canadensis* cluster [12, 34–36], these primers were expected to work.

### DNA sequence assembly

We used NOVOPlasty (https://github.com/ndierckx/NOVOPlasty) to assemble consensus sequences [37]. MAFFT (https://mafft.cbrc.jp) was used to align multiple sequences [38]. The sequences were manually verified using BioEdit 7.2.1 (https://bioedit.software.informer.com) [39]. Two different datasets were used in this study: (1) near-complete/complete mtDNA of all samples (dataset A); (2) major genes of mtDNA of all samples (dataset B).

### Phylogenetic analysis and comparison tests

We used IQ-TREE (version 1.6.12) to construct a maximum-likelihood tree with the near-complete/complete mtDNA sequences (dataset A) to identify the genotypes and species of the *Echinococcus* spp., and ModelFinder was used to identify the best-fit nucleotide substitution models [40, 41]. A bootstrap value of 1000 replicates was used to test the robustness of the phylogenetic tree. A clade was supported if there were three or more samples (including the reference sequence) and the approximate likelihood-ratio test SH-aLRT ≥ 80% and UFboot ≥ 95% [42, 43]. The mitochondrial sequence data of *Versteria mustelae* (AB732957) were used as an outgroup of the genus *Echinococcus* [44]. The other reference sequences included *E. granulosus* G1 (AB786664), *E. granulosus* G3 (KJ559023); *E. equinus* G4 (AB786665); *E. ortleppi* G5 (AB235846); G6 of the *E. canadensis* cluster (AB208063), G7 of the *E. canadensis* cluster (AB235847), *E. canadensis* G8 (AB235848), *E. canadensis* G10 (AB745463); *E. felidis* (AB732958); *E. multilocularis* (AB018440.2); *E. shiquicus* (AB208064); *E. oligarthra* (AB208545); and *E. vogeli* (AB208546). The analysis was tested at least twice with the same dataset to verify the consistency of the results. After the species and genotypes were identified with near-complete/complete mtDNA, the same procedures were carried out with the major mitochondrial genes used in the literature, including *cox*1, *nad*1, *nad*2, *nad*5, and *atp*6 (dataset B), to test whether, using the key genes, the species and genotypes could be identified successfully.

After that, non-parametric two-tailed Wilcoxon-Mann-Whitney tests were performed to check whether the average cyst size of the different species/genotypes of CE infection was significantly different (*P* < 0.05 was considered significant). Only those genotypes with over five samples were included. Spearman’s correlation (*r*) was used to assess the correlations between age and the average cyst size (*P* < 0.05 was considered significant).

### Phylogenetic networks and genetic variability

The phylogenetic networks were built with PopART (http://popart.otago.ac.nz) [45]. Arlequin 3.5.2.2. was used to calculate the neutrality indices Tajima’s *D* and Fu’s Fs among all the G1 samples [46–48]. Other population diversity indices were calculated with DnaSP v6.12.03 [49].

### Bayesian phylogeographic analysis

To understand the phylogeographical diffusion pattern, especially how G1 spread to TAR in China, we analyzed our G1 samples together with the 212 G1 samples from Kinkar et al. through a Bayesian discrete phylogeographic method [13, 50]. Using the Bayesian stochastic search variable selection (BSSVS), we reconstructed the phylogeographic diffusion process through a Bayes factor (BF) test [50]. The procedures followed the Beast Tutorial titled “Phylogeographic diffusion in discrete space” (https://beast.community/workshop_discrete_diffusion—accessed in March 2021). The sampling dates of our samples used the month of the surgery when the cysts were taken out. As no indication of the other samples’ collection dates was available, we used the date when the manuscript with the sample was first submitted. The analysis was performed with BEAST v1.10.4 using the BEAGLE library [51, 52]. The best fit model was chosen with ModelFinder [40]. Under the corrected Akaike information criterion (AIC), the best model was GTR + F + I + G4. MCMC chains were run for 100 million states, sampling every 10,000 steps with 10% burn-in, after which, Tracer v1.7.1 was used to check the effective sampling size (ESS) of estimates [51, 53]. The MCC tree was produced with TreeAnnotator v1.10.4 and visualized with SpreaD3 v0.9.7.1 [51, 54]. The combination of the runs was carried out with LogCombiner v1.10.4 [51]. BF and posterior probabilities were generated with SpreaD3 v0.9.7.1 [54]. Only routes which could meet BF ≥ 3 and the node posterior probabilities ≥ 0.5 were interpreted as supported [50, 55]. The map was drawn with R version 4.1.1.

## Results

### Phylogenetic identification of the species and genotypes

**Fig. 1:**
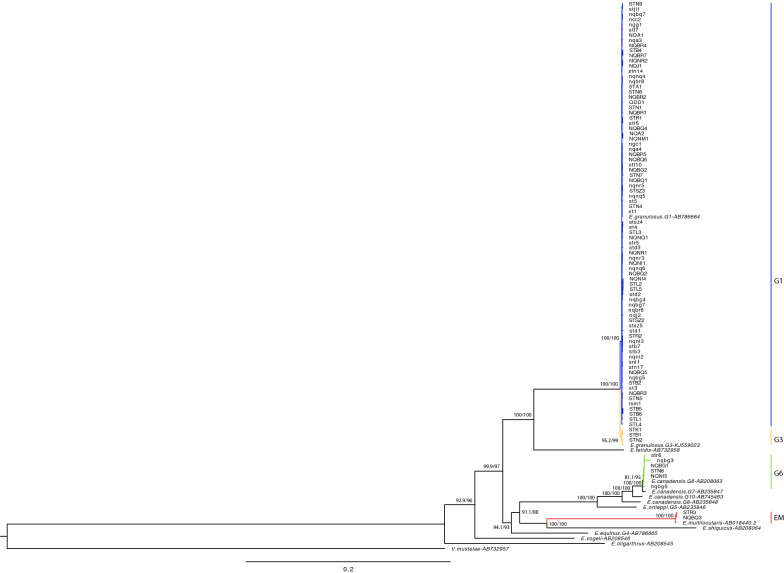
Phylogenetic tree of *Echinococcus* spp. and genotypes with a maximum-likelihood approach using the near-complete/complete mtDNA sequences

A total of 92 near-complete/complete mtDNA sequences were successfully obtained from 92 patients’ samples, among which 50 samples were sequenced directly, and 42 samples were sequenced after PCR amplification. Of the 13 PCR primer pairs published by Laurimae et al., 12 worked (the E9 primer pair failed) [12]. Phylogenetic identification of the sequences is shown in Fig. 1, which used the near-complete/complete mtDNA from all the cysts samples (dataset A) and the key references of *Echinococcus* spp. and genotypes. The major mtDNA genes of all the samples (dataset B) were used to construct the maximum-likelihood trees together with the key reference sequences’ genes, which are given in Additional file 1: Figure S1, Additional file 2: Figure S2, Additional file 3: Figure S3, Additional file 4: Figure S4, Additional file 5: Figure S5.

As Fig. 1 shows, the analysis using near-complete/complete mtDNA sequences divided the samples into four genotypes in three species. Genotype G1 from *E. granulosus* (s.s.) accounted for the majority (*n* = 81, 88.04%), followed by G6 of the *E. canadensis* cluster (*n* = 6, 6.52%), G3 from *E. granulosus* (s.s.) (*n* = 3, 3.26%) and *E. multilocularis* (*n* = 2, 2.17%). Using dataset B from the nucleotide sequences of major mitochondrial genes (*cox*1, *nad*1, *nad*2, *nad*5, and *atp*6) (Additional file 1: Figure S1, Additional file 2: Figure S2, Additional file 3: Figure S3, Additional file 4: Figure S4, Additional file 5: Figure S5), G1/G3 and G6/G7 could not be successfully separated, except in the topology made with *nad*5, which could separate G1/G3. The majority of G1 samples were 12,027 bp in length, with four short sequences (NGG1, LSM1, NQNQ4, and STN7) as 11,007 bp, and the main gene affected was *nad*5; thus, Additional file 4: Figure S4 was made with only 88 *nad*5 sequences together with the reference sequences. The lengths of G3 and G6 were 13,702 bp and 11,603 bp, respectively. The length of *E. multilocularis* was 13,738 bp.

### Participants’ demographic characteristics and clinical presentation

A summary of the patients’ demographics (age, sex, and residency), sample information (endocyst/cyst fluid), cyst information (number, location, average size, WHO Informal Working Group on Echinococcosis (WHO-IWGE) cyst classification [56]), and qPCR results is given in Table 1. Detailed information including genotypes and species results (obtained from the maximum-likelihood method) of all the participants is given in Additional file 6: Table S1.

**Table 1:** Summary of patient demographics, sample information, and cyst information

	Number	%
Age (years)		
Maximum	61	NA
Minimum	5	NA
Mean	31 (SD 14.6)	NA
Sex		
Male	30	32.61
Female	62	67.39
Sample analyzed		
Cyst fluid	5	5.43
Endocyst	87	94.57
Cyst location		
Liver only	83	90.22
Another organ only	3	3.26
Liver + another organ	5	5.43
Missing	1	1.09
Average cyst size (*n* = 91)		
Large (> 10 cm)	26	28.57
Medium (5–10 cm)	49	53.85
Small (< 5 cm)	16	17.78
Cyst stage (*n* = 90)		
≥ 1 active cyst (CE1 and CE2)	52	57.78
Transitional CE3	15	16.67
Mostly inactive (CE4 and CE5)	20	22.22
Unknown	3	3.33
Missing	1	2.17
qPCR		
G1	79	85.87
*E. multilocularis*	1	1.09
*E. multilocularis* and G1	2	2.17
Not performed	7	7.61
Failed	3	3.26

Individual patient details are given in Additional file 6: Table S1

*NA* not applicable, *SD* standard deviation

Most patients were females (67.39%). All except three patients were undergoing the surgery for the first time. Nearly all the cysts were from hepatic lesions (95.65%). Five patients had lesions in the liver and one more region, including two in the pelvic cavity, two in the spleen, and one in the abdomen. Most of the hepatic lesions were in the right liver only (73.86%), while 14.77% affected the left liver only.

All G3 and *E. multilocularis* infected patients’ cysts were found in the right liver only, and in 66.67% of the G6 patients were found in the right liver only. All the G3 cysts were classified as CE1, and 85.71% of the G6 cysts (six out of seven) were classified as CE3, including two cysts (both CE3) from one patient. In comparison, 28.40% of G1 patients had at least one CE1, 18.52% had at least one CE3, and 33.33% G1 patients had at least one CE2. Some G1 patients' cysts were CE4 and/or CE5.

Most of the cysts/pseudocysts were > 5 cm (82.42%). The average size was 8.4 cm (95% CI 7.7–9.1) for all the cysts and G1, 11.2 cm for G3, and 6.3 cm for G6 (95% CI 3.8–8.8) and 9.5 cm for the pseudocysts caused by *E. multilocularis*. As too few samples were found in G3, no further comparison was made for them. A non-parametric two-tailed Wilcoxon-Mann-Whitney test was carried out to test whether the average size of G6 would be the same as that of G1 with the null hypothesis. Comparing the cyst sizes of G6 of the *E. canadensis* cluster and *E. granulosus* G1 identified *P* = 0.084, meaning the null hypothesis was likely, with no difference between the cyst sizes caused by the two genotypes. The mean ages of G1 and G6 patients were 31.3 (95% CI 27.9–34.6) and 24.7 (95% CI 17.2–32.2) years, respectively (two-sided Wilcoxon-Mann-Whitney test *P* = 0.361), meaning the two groups of patients’ ages were not statistically different. Spearman's correlation between age and the average CE cyst size showed a positive correlation (*r*_s_ = 0.3195, *P* = 0.0039).

Using ultrasound, only one AE case (NQBQ3) was identified successfully, but no conclusive diagnosis was made for the other AE case (STR3). In contrast, the qPCR method only identified one AE (STR3) accurately, and the other AE case (NQBQ3) was identified as “*E. multilocularis* and G1” infection, which was the same result as for one G1 sample (NQBQ4). All G3 and G6 samples were classified as G1 according to the qPCR result.

### Spatial distribution of the genotypes identified

**Fig. 2:**
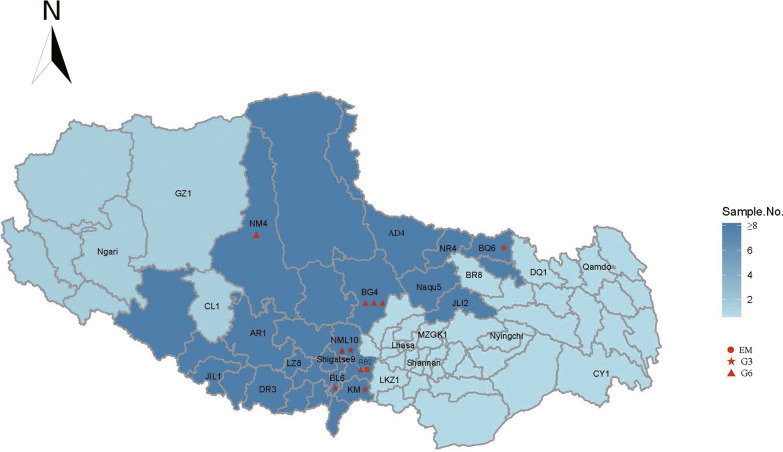
DSistribution of *Echinococcus* spp. and genotypes in TAR in this study. The numbers next to the county abbreviations as the number of G1 samples identified in that county. Other genotypes numbers were marked in red

All patients were Tibetans living in TAR throughout their lives. The spatial distribution of the species and genotypes can be found in Fig. 2.

As shown in Fig. 2, the patients were from all seven prefectures, with most from Naqu and Shigatse. The two AE cases were from Naqu and Shigatse. Most of the G6 cases (four out of six) were from Naqu, and all G3 cases were from Shigatse.

### Phylogenetic networks of the *E. granulosus* (s.s.) samples and references

**Fig. 3:**
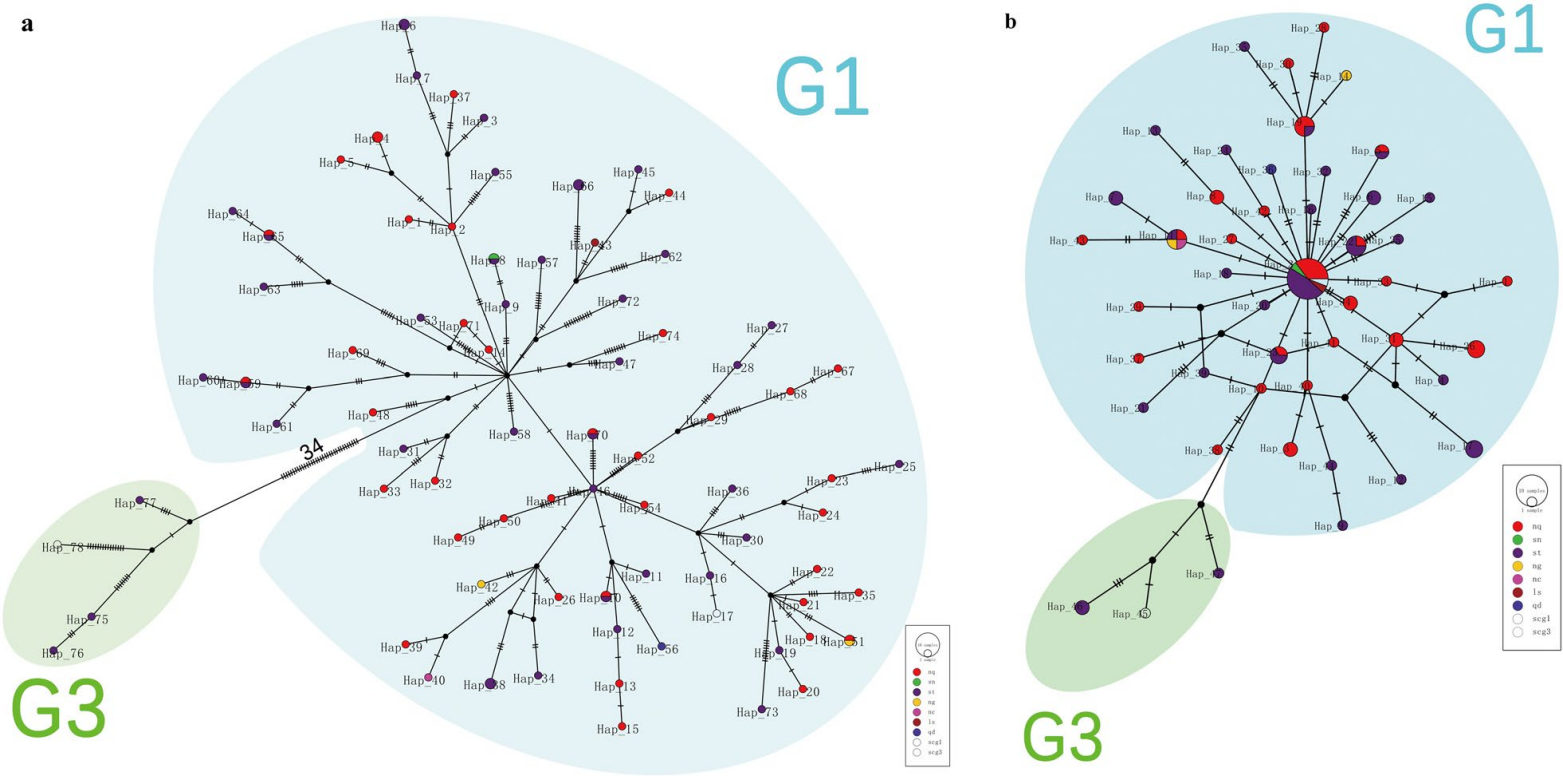
Median-joining network of *E. granulosus* (s.s.) samples using near-complete mtDNA (11,007 bp) and *cox*1 (1674 bp). **a** Network of the *E. granulosus* (s.s.) samples using near-complete mitochondrial sequences. **b** Network of the *E. granulosus* (s.s.) samples using the *cox*1 gene. The circle size is proportional to the number in the haplotypes, and every notch indicates a mutation between the two haplotypes. The black dots mean the presumed missing median vectors

As shown in Fig. 3, the phylogenetic networks were made using near-complete mtDNA (11,007 bp) and *cox*1 (1674 bp) of the mtDNA sequences from *E. granulosus* (s.s.) samples with reference G1 (AB786664) and G3 (KJ559023) [57, 58].

Figure 3a shows that using the near-complete G1 and G3 sequences, 78 haplotypes were formed, and the pattern went radially from the center. There were two major G1 clusters of sequences, with a branch of G3 farthest from the root by 34 mutations away from the G1 cluster. Using G3 reference KJ559023 and our G3 samples as the outgroup, Hap_14/NQBR6 from Naqu was closest to the root. Equally far, Hap_46/STN14 from Nanmulin in Shigatse was the center of the other cluster. The reference G1 sequence AB786664 or Hap_17 was three mutations from the center Hap_46/STN14. The location of STN14, Nanmulin, was bordering Naqu. The G1 samples from Shigatse and Naqu were interwoven, with no obvious geographical clustering.

Figure 3b, which is the network of the *E. granulosus* (s.s.) samples using the *cox*1 gene, shows that our samples formed 47 haplotypes. Using G3 as the outgroup (Hap_45/KJ559023, Hap_46/STK1&STB1, and Hap_47/STN2), an expanding trend was observed, though with reduced resolution compared with Fig. 3a. A major haplotype at the center Hap_2 included 17 samples (8 from Shigatse, 6 from Naqu, 1 from Shannan and Lhasa each, and G1 reference AB786664). G3 *cox*1 reference from KJ559023 was five mutations away from the root, clustered with our G3 samples.

### Phylogenetic relationship of the G1 samples of TAR and neighboring provinces

**Fig. 4:**
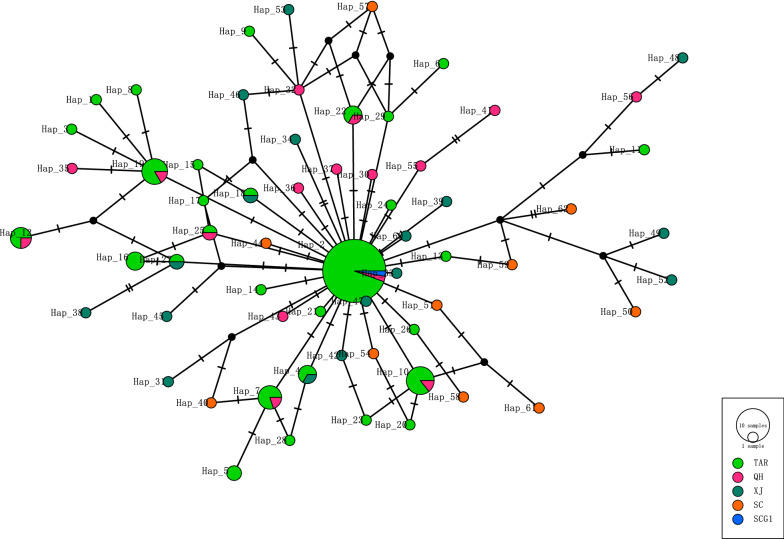
Median-joining network using partial *cox*1 gene (789 bp) from *E. granulosus* (s.s.) G1 samples and other G1 samples (*n* = 43 haplotypes) from three neighboring provinces [9] and also the G1 reference sequence AB786664 (SCG1)

To understand the relationship with other neighboring provinces, we added G1 samples from Qinghai, Xinjiang, and Sichuan downloaded from NCBI based on a previous paper [9] to draw the network, shown in Fig. 4. As mentioned, previous research mainly used shorter fragments such as partial *cox*1, *cox*1, or *nad*1; we could only use shorter fragments to draw the network with the samples from the neighboring provinces. We also added the reference sequence of G1 (AB786664).

A star pattern was identified, and the major haplotype Hap_2 included 34 samples from TAR, 1 sample from Qinghai, and the common haplotype G01 (reference AB786664–scg1). Many haplotypes were shared by the samples from TAR and Qinghai.

As many of our G1 haplotypes were shared with Qinghai samples, we calculated the Fst of the G1 population from the four provinces in China, shown in Table 2.

**Table 2:** Pairwise fixation index (Fst) values between the G1 samples from TAR and the neighboring provinces using 789 bp of mtDNA

	TAR	Qinghai	Xinjiang	Sichuan
TAR	–			
Qinghai	0	–		
Xinjiang	0.05*	0.01	–	
Sichuan	0.09*	0.03	0	–

*Significant *P* value (< 0.05)

As Table 2 shows, Fst indicated that there was no differentiation in sub-populations between TAR and Qinghai. Moderate differentiation was found between TAR and Sichuan (Fst = 0.09) and Xinjiang (Fst = 0.05).

### Population diversity and neutrality indices of the G1 samples

Population diversity and neutrality indices of the G1 samples were calculated based on the near-complete sequences and the key genes. The results are shown in Table 3.

**Table 3:** Diversity and neutrality values of the G1 samples included in this study using different lengths/genes of mtDNA sequences

	Diversity				Neutrality	
	*n*	Hn	Hd ± SD	*π* ± SD	*D*	Fs
Total						
Samples (12,027 bp)	77^a^	69	0.997 ± 0.003	0.00112 ± 0.00005	− 2.57989^c^	− 24.26656^c^
*Cox*1 (1674 bp)	81	44	0.952 ± 0.016	0.00183 ± 0.00015	− 2.48397^c^	− 26.44792^c^
Partial *Cox*1 (789 bp)	81	29	0.815 ± 0.043	0.00208 ± 0.00022	− 2.19766^c^	− 27.87330^c^
*Nad*1 (894 bp)	81	13	0.571 ± 0.062	0.00087 ± 0.00014	− 2.04308^b^	− 10.53838^c^
*Nad*2 (882 bp)	81	25	0.702 ± 0.058	0.00129 ± 0.00017	− 2.47233^c^	− 29.01818^c^
*Nad*5 (1022 bp)	77^a^	23	0.776 ± 0.047	0.00132 ± 0.00015	− 2.26119^c^	− 22.93365^c^
*Atp*6 (513 bp)	81	10	0.642 ± 0.033	0.00156 ± 0.00015	− 1.44069	− 5.67056^b^

*n* number of samples examined, *Hn* number of haplotypes, *Hd* haplotype diversity, *pi (π)* nucleotide diversity, *D* Tajima’s *D*, *Fs* Fu’s Fs

^a^We failed to obtain partial *nad*5 gene sequences from four G1 samples

^b^*P* < 0.01

^c^*P* < 0.001

As shown in Table 3, among the 81 G1 samples, the haplotype diversity (Hd) index was the highest with the near-complete sequences. The second highest Hd index and the number of haplotypes were with *cox*1. Both near-complete mtDNA and most of the genes (except *atp*6) showed significant negative values, which indicated the deviation from neutrality was significant.

### Bayesian phylogeographic analysis of the G1 samples

**Fig. 5:**
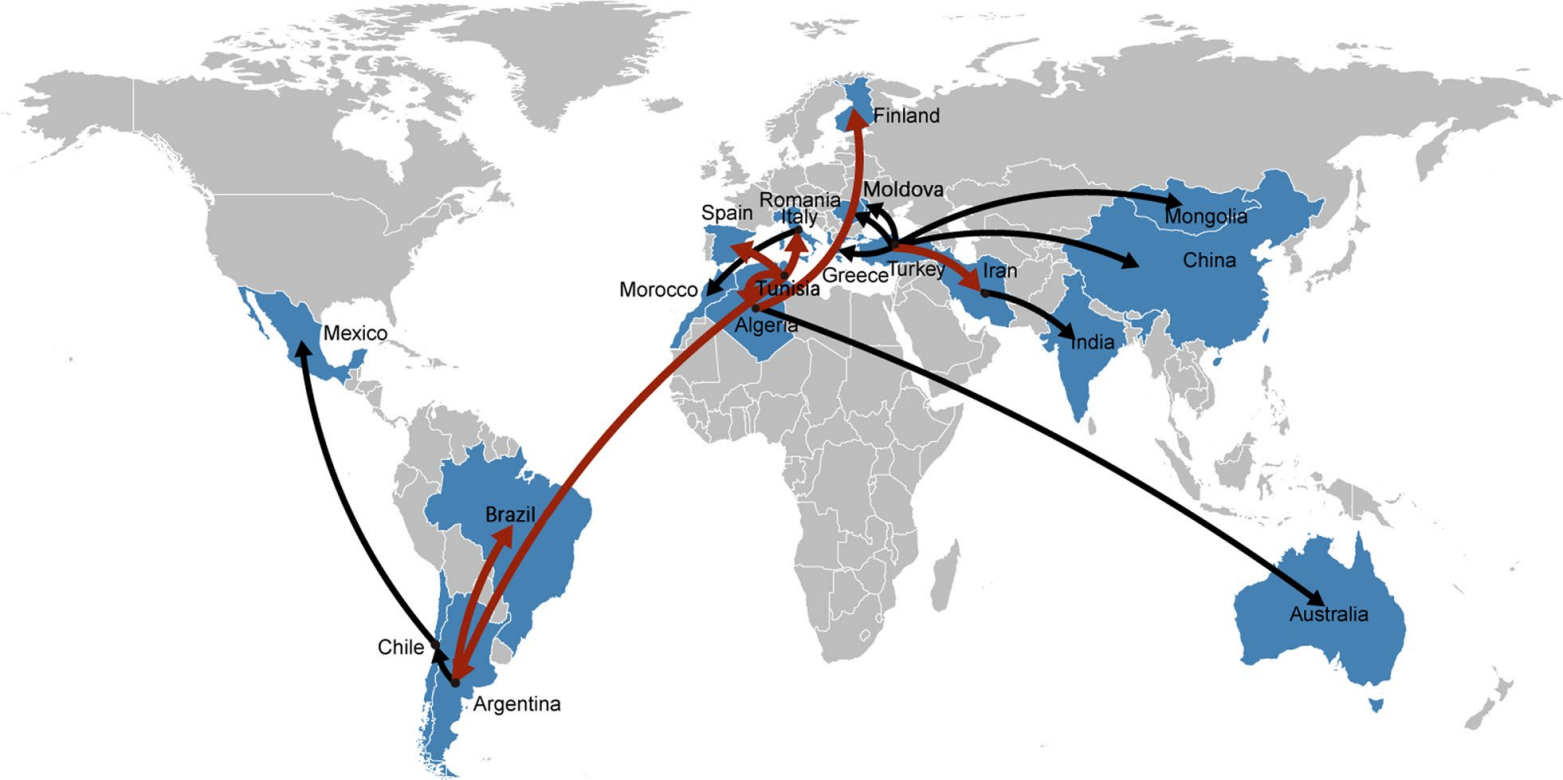
Well-supported spatial diffusion pathways of the spread of *E. granulosus* (s.s.) genotype G1. The BSSVS analysis was carried out and redrawn with 293 G1 samples (81 G1 samples from this study from TAR in China and 212 previous published G1 samples from 22 countries in Kinkar et al. [13]—12,385 bp of mtDNA). Black lines represent the routes with BF > 20, and red lines represent the routes with BF > 300

To understand the phylogeographical diffusion routes and how G1 spread to China, we analyzed our G1 samples together with the 212 G1 samples from Kinkar et al. through a Bayesian discrete phylogeographic method [13, 50]. Based on the selection criteria, diffusion routes with BF ≥ 3 and node posterior probabilities ≥ 0.5 were supported. The results are shown in Fig. 5.

As shown in Fig. 5, 17 well-supported spatial routes were identified. Ten routes had BF > 20 and seven BF > 300. Six out of the 17 routes were originated from Turkey, including the one spreading to China (BF > 53, posterior probabilities > 0.7). The six countries whose routes originated from Turkey included Iran, Greece, Mongolia, China, Moldova, and Romania (BF and posterior probability in decreasing order). Four routes originated from Tunisia, namely Algeria, Italy, Argentina, and Spain (BF and posterior probability in reducing order).

## Discussion

Western China is an endemic region of both CE and AE, and TAR ranks top in human echinococcosis prevalence among the western provinces of China [24, 28, 59]. Although the prevalence of echinococcosis was explored [28], the causative agents of human infection have not yet been fully understood in TAR. Previous molecular studies on the Tibetan Plateau often included samples from animals; even when samples from humans were involved, they were mainly patients from Qinghai or Sichuan Provinces, with few patients from TAR; moreover, previous studies were using short fragments of the mtDNA with limited phylogenetic resolution [5, 9, 25–27].

In this research, using the near-complete/complete mitochondrial sequences obtained from the cysts of 92 echinococcosis patients in a designated hospital, the exact *Echinococcus* species and genotypes infecting humans all over TAR were clarified to be three species and four genotypes: *E. granulosus* (s.s.) G1 accounting for the majority, followed by G6 of the *E. canadensis* cluster, *E. granulosus* (s.s.) G3, and *E. multilocularis*. As far as we know, this is the first comprehensive molecular investigation of *Echinococcus* species and genotypes infecting humans based on near-complete/complete mtDNA sequences. The genetic variability was explored, and the population expansion trend of G1 was identified. For the first time, we also identified the possible origin of the G1 in TAR, which was probably from Turkey, in line with Kinkar et al., who reported Turkey or the Middle East (the origin of livestock domestication) as the origin of G1 [13, 60]. However, the analysis, as highlighted by Kinkar et al., could be influenced by the unbalanced samples obtained [13]. The complete mtDNA database was also enriched through this study. Previously, in NCBI, there was only one complete genome of *E. multilocularis* (AB018440.2, 13,738 bp), which was from a naturally infected vole from Hokkaido and maintained through Mongolian gerbils [61]. Two complete *E. multilocularis* mtDNA sequences (13,738 bp) obtained from Tibetan patients were added through this study. Similarly, there was only one complete mtDNA of *E. granulosus* G3 stored in NCBI (KJ559023.1 13,702 bp), which was from a Sichuan patient [58]. Three mtDNAs of *E. granulosus* G3 sequences (13,702 bp) were added through this study.

Using near-complete/complete mitochondrial sequences to identify the exact species and genotypes of the echinococcosis infection was feasible, providing a better phylogenetic resolution and accuracy than commonly used short genes. Understanding of the species and genotypes is essential for targeted diagnostic tool development and control strategies [8, 62]. For example, Yang et al. reported only G1 was identified in the Tibetan communities in Sichuan Province; thus, the key control method would be the Eg95 vaccine together with dog anthelmintic treatment [63]. However, this method may not be sufficient in areas with more *Echinococcus* species and genotypes, where more monitoring of the respective hosts, more hygiene education, and dog treatment are needed [63]. The *Echinococcus* species or genotypes identified in human patients could all be due to eggs produced by adult *Echinococcus* spp. parasites in the definite hosts, which indicates the relationships between the pathogens of humans and animals. More *Echinococcus* species and genotypes identified in an area mean more animals involved in the transmission cycle, and the targeted control in this region should be more than targeting sheep and dogs. Though human pathogens are not involved in the transmission of echinococcosis at all, given the sources of human pathogens could all be animals, it is possible to make phylogenetic trees with samples from humans and animal sources. With near-complete/complete mtDNA, the closely related genotypes such as G1/G3 and G6/G7 were differentiated, which could not be achieved using key genes including *cox*1, *nad*1, *nad*2, and *atp*6, as demonstrated in the phylogenetic trees, but it was possible to differentiate G1/G3 with *nad*5, which confirmed the previous study finding of Kinkar et al. [64].

Ultrasound only diagnosed one AE patient accurately. QPCR only identified one AE patient accurately, and the other AE patient had “*E. multilocularis* and G1” infection, which was the same result as in one G1 patient. The qPCR method could only identify the three species as indicated in the reference, namely *E. granulosus* (G1), *E. multilocularis*, and *E. shiquicus* [31]; closely related species/genotypes may be identified as one of the three. Near-complete/complete mtDNA could be considered in areas with multiple species and genotypes such as TAR to identify the accurate species and genotypes among patients who need surgery to remove the lesion when the differential diagnosis such as imaging or qPCR is not conclusive. No *E. shiquicus* infection was identified with either qPCR or mtDNA method, which confirmed that humans were probably insusceptible to *E. shiquicus*, a new species found in the Tibetan Plateau only, despite the concern of its potential, given dog infection of *E. shiquicus* was reported [65]. Besides, near-complete/complete mitochondrial sequences of patients could serve as baseline data to monitor the *Echinococcus* species and genotype prevalence changes, mtDNA mutations, and trends of *Echinococcus* spp. population.

Like many other areas in the world, *E. granulosus* (s.s.), especially G1, accounted for most of our samples [4, 9, 63, 66, 67]. Using the *cox*1 gene, Heath et al. reported the samples obtained from yaks from Tibetan Plateau as G1 genotype infection, but no protoscoleces were identified in their cysts [67]. Due to the short target genes used, most of the previous studies could not differentiate the genotypes within *E. granulosus* (s.s.) with confidence. With near-complete/complete mtDNA, three G3 samples were identified, all from Shigatse. Interestingly, G3 was reported as the dominant genotype infecting humans in Pakistan and North India [11, 15], which bordered TAR, especially Shigatse directly or via Nepal. There were limited molecular studies on causative species and genotypes of echinococcosis patients in Nepal, but it was reported that 5% of the water buffalos examined were found to have hydatid cysts, and G3 was previously called the buffalo strain [14, 68]. Though limited in the number of G3 samples, we found all were active CE1. Further studies in this region might help us to explore the characteristics and ultrasound presentation of G3 infection.

Our identified genotypes G1, G3, and G6 are in line with the genotypes identified in the study with animal samples (sheep and yaks) in TAR [27]. Using *nad*1 and *nad*5 genes through BLAST comparison, G1, G3, and G6 were found to be the causative agents of cysts in the animal samples from Shigatse and Lhasa, and the authors confirmed the two G6 samples from Zhongba county in the west of Shigatse with complete G6 mtDNA [27]. The two G6 samples in our study were from Nanmulin and Renbu in the east of Shigatse, and the other four G6 samples were from Naqu. The geographical distribution of G6 was wider than in the previous reports, and further molecular studies in TAR could improve our understanding of G6. The reason behind the wide distribution of G3 and G6 in TAR is to be explored.

Molecular identification of the causative agents is not only a prerequisite to understanding the epidemiology and pathology of *Echinococcus* spp. but also essential to formulate targeted control measures [4, 7, 8]. Our study, though preliminary, improved our understanding of the ultrasound characteristics of G1, G3, and G6. G3 infections were large active CE1, and G6 infections were mostly transitional CE3 with smaller cysts. The average age of patients infected by G6 seemed younger at 24.7 (95% CI 17.2–32.2) compared to G1 patients at 31.3 (95% CI 27.9–34.6) years, but no significant difference was found. Though it was reported in Mongolia that all pediatric cases were infected with *E. canadensis*, especially G6/G7 of the *E. canadensis* cluster [6], in our study, 90% of the children were infected with G1, with only two children infected with G6. It was hypothesized that the lack of adult cases infected with G6/G7 in the Mongolia study could be explained in two opposite directions: *E. canadensis* infection could be mild and spontaneously healed among children or lead to early death of the children infected with G6/G7 [4]. Our study supports the mild G6 hypothesis, given that (i) most of the G6 cysts were transitional CE3; (ii) most of the G6 cysts were small; (iii) all of the G6 patients were > 14 years old, with 66.7% > 27 years old; (iv) most of the G6 patients came to the hospital because of detection of the cyst by ultrasound in either regular body check-ups or echinococcosis screening programs instead of symptoms. As there were no symptoms, even after ultrasound positive results of echinococcosis, the patients waited 5–10 months before coming to the hospital to undergo surgery. This was in line with the findings of Schneider et al. among the patients in Europe [10]. Due to the limited sample size, this mild hypothesis of G6 needs further verification in larger scale studies with molecular markers.

Except for *atp*6, all the genes of G1 studied inferred significant deviation from neutrality. The expansion trend and a possible recent bottleneck event were confirmed in the region as hypothesized by Nakao et al. [9]. The *nad*5 gene was not complete in four samples sequenced after PCR and the G1 samples were only near-complete, probably because the *nad*5 gene was located next to the tandem repeat region [69], which made it difficult to carry out PCR; thus, for G1 samples, it was hard to obtain the full mtDNA sequences. The other possible reason could be that these four samples were not as good as the other samples and the PCR primers for *nad*5 failed. Of the 13 PCR primer pairs published before, 12 pairs worked; the E9 primer pair was specially designed for G6/G7 [12], which might not be suitable for G1/G3 samples. For samples with low-quality and low-concentration DNA, these 12 primer pairs could be used to amplify DNA to obtain near-complete mtDNA, but the targeted region of the E9 primer pair should be improved by considering more genotype sequences such as G1/G3. Our team is exploring this direction.

Only one hospital specialized in hepatic cyst removal was selected as the study site, thus limiting the cysts/pseudocysts from other organs and regions, which might have limited the generalizability of our study. It was hypothesized that different *Echinococcus* species and genotypes might have different organotropism, for example, G6 might exhibit more brain tropism than G1 [70]. In this research, the hospital only examined the brain if some symptoms/indicators possibly involved the brain. Even with this limitation, patients from all seven prefectures were included, and a variety of species and genotypes infecting humans were identified; thus, more cyst/pseudocyst samples from more organs and hospitals would probably increase the diversity identified.

Another limitation was that no follow-up of the patients after the surgery was performed; thus it was not known if there was any difference between the prognoses of the patients infected by different *Echinococcus* species and genotypes. The lack of follow-up also limited the possible application of mtDNA sequences to differentiate between re-infection and relapse. It is hypothesized that if the mtDNA sequences obtained from the new cysts/pseudocysts are identical to the corresponding patients’ mtDNA identified this time, it would probably be due to relapse, but if the mtDNA sequences obtained from the new cysts/pseudocysts are different from the corresponding patients’ mtDNA identified this time, it would probably be re-infection. It is believed that with the near-complete mitochondrial sequence records and mtDNA sequences obtained from the patients’ new cyst, the re-infection or relapse could be differentiated. A larger scale study design with follow-up should be planned in the future in this direction.

## Conclusions

We conducted a comprehensive molecular investigation of *Echinococcus* species and genotypes infecting humans throughout TAR based on near-complete/complete mtDNA sequences. We found patients infected by three *Echinococcus* species and four genotypes including *E. granulosus* (s.s.) G1 and G3, G6 of the *E. canadensis* cluster, and *E. multilocularis*. The genetic variability was explored, and the population expansion trend of G1 was identified. For the first time, we also identified the possible origin of the G1 in TAR in China. The findings also enriched the long mtDNA database of *Echinococcus* spp. and added two complete *E. multilocularis* mtDNA sequences from humans. The findings improved our knowledge of echinococcosis infecting humans in TAR, a region heavily affected by echinococcoses, which would help to refine the targeted echinococcosis control measures and serve as valuable baseline data for monitoring the *Echinococcus* species and genotype prevalence changes, mtDNA mutations, and trends of the *Echinococcus* population in the region.

## Supplementary Information

**Additional file 1: Figure S1.** Phylogenetic tree of *Echinococcus* spp. and genotypes with a maximum-likelihood approach using the cox1 gene sequences (92 from this study and 14 references).

**Additional file 2: Figure S2.** Phylogenetic tree of *Echinococcus* spp. and genotypes with a maximum-likelihood approach using the nad1 gene sequences (92 from this study and 14 references).

**Additional file 3: Figure S3.** Phylogenetic tree of *Echinococcus* spp. and genotypes with a maximum-likelihood approach using the nad2 gene sequences (92 from this study and 14 references).

**Additional file 4: Figure S4.** Phylogenetic tree of *Echinococcus* spp. and genotypes with a maximum-likelihood approach using the nad5 gene sequences (88 from this study and 14 references).

**Additional file 5: Figure S5.** Phylogenetic tree of *Echinococcus* spp. and genotypes with a maximum-likelihood approach using the atp6 gene sequences (92 from this study and 14 references).

**Additional file 6: Table S1.** Detailed information of the patients and the *Echinococcus* spp. and genotypes identified (obtained from the maximum-likelihood method).

## Data Availability

The data reported in this study are stored in the CNGB Nucleotide Sequence Archive (CNSA: https://db.cngb.org/cnsa with accession number CNP0001078).

## Abbreviations

AE: Alveolar echinococcosis
CE: Cystic echinococcosis
DALYs: Disability-adjusted life years
*E. canadensis*: *Echinococcus canadensis*
*E. granulosus* (s.s.): *Echinococcus granulosus* (s.s.)
*E. multilocularis*: *Echinococcus multilocularis*
ELISA: Enzyme-linked immunosorbent assay
FAO: Food and Agriculture Organization
Hd: Haplotype diversity
mtDNA: Mitochondrial DNA
NTDs: Neglected tropical diseases
PCR: Polymerase chain reaction
TAR: Tibet Autonomous Region
WHO: World Health Organization
WHO-IWGE: The WHO Informal Working Group on Echinococcosis

## References

[1] World Health Organization (2012). Accelerating work to overcome the global impact of neglected tropical diseases: a roadmap for implementation: executive summary.

[2] McManus DP, Gray DJ, Zhang W, Yang Y (2012). Diagnosis, treatment, and management of echinococcosis. BMJ.

[3] Food and Agriculture Organization of the United Nations/World Health Organization. Multicriteria-based ranking for risk management of food-borne parasites. Microbiological Risk Assessment Series No. 23. Rome: FAO/WHO; 2014.

[4] Ito A, Nakao M, Lavikainen A, Hoberg E (2017). Cystic echinococcosis: future perspectives of molecular epidemiology. Acta Trop.

[5] Alvarez Rojas CA, Romig T, Lightowlers MW (2014). *Echinococcus granulosus* sensu lato genotypes infecting humans—review of current knowledge. Int J Parasitol.

[6] Ito A, Dorjsuren T, Davaasuren A, Yanagida T, Sako Y, Nakaya K (2014). Cystic echinococcoses in Mongolia: molecular identification, serology and risk factors. PLoS Negl Trop Dis.

[7] Thompson RCA, McManus DP, Eckert J, Gemmell M, Meslin F-X, Pawlowski Z (2001). Aetiology: parasites and life-cycles. WHOO/OIE manual on echinococcosis in humans and animals: a public health problem of global concern.

[8] Wen H, Vuitton L, Tuxun T, Li J, Vuitton DA, Zhang W (2019). Echinococcosis: advances in the 21st century. Clin Microbiol Rev.

[9] Nakao M, Li T, Han X, Ma X, Xiao N, Qiu J (2010). Genetic polymorphisms of *Echinococcus* tapeworms in China as determined by mitochondrial and nuclear DNA sequences. Int J Parasitol.

[10] Schneider R, Gollackner B, Schindl M, Tucek G, Auer H (2010). *Echinococcus canadensis* G7 (pig strain): an underestimated cause of cystic echinococcosis in Austria. Am J Trop Med Hyg.

[11] Muqaddas H, Mehmood N, Arshad M (2020). Genetic variability and diversity of *Echinococcus granulosus* sensu lato in human isolates of Pakistan based on cox1 mt-DNA sequences (366bp). Acta Trop.

[12] Laurimae T, Kinkar L, Romig T, Omer RA, Casulli A, Umhang G (2018). The benefits of analysing complete mitochondrial genomes: deep insights into the phylogeny and population structure of *Echinococcus granulosus* sensu lato genotypes G6 and G7. Infect Genet Evol.

[13] Kinkar L, Laurimae T, Acosta-Jamett G, Andresiuk V, Balkaya I, Casulli A (2018). Global phylogeography and genetic diversity of the zoonotic tapeworm *Echinococcus granulosus* sensu stricto genotype G1. Int J Parasitol.

[14] Bowles J, Blair D, McManus DP (1992). Genetic variants within the genus *Echinococcus* identified by mitochondrial DNA sequencing. Mol Biochem Parasitol.

[15] Sharma M, Sehgal R, Fomda BA, Malhotra A, Malla N (2013). Molecular characterization of *Echinococcus granulosus* cysts in North Indian patients: identification of G1, G3, G5 and G6 genotypes. PLoS Negl Trop Dis.

[16] Zhang LH, Chai JJ, Jiao W, Osman Y, McManus DP (1998). Mitochondrial genomic markers confirm the presence of the camel strain (G6 genotype) of *Echinococcus granulosus* in north-western China. Parasitology.

[17] Bart JM, Abdukader M, Zhang YL, Lin RY, Wang YH, Nakao M (2006). Genotyping of human cystic echinococcosis in Xinjiang, PR China. Parasitology.

[18] Zhang T, Yang D, Zeng Z, Zhao W, Liu A, Piao D (2014). Genetic characterization of human-derived hydatid cysts of *Echinococcus granulosus* sensu lato in Heilongjiang Province and the first report of G7 genotype of *E. canadensis* in humans in China. PLoS ONE.

[19] Ali V, Martinez E, Duran P, Seláez MA, Barragan M, Nogales P (2020). *Echinococcus granulosus* sensu stricto, *Echinococcus ortleppi*; and *E. intermedius* (G7) are present in Bolivia. Parasitology.

[20] Lymbery AJ (2017). Phylogenetic pattern, evolutionary processes and species delimitation in the genus *Echinococcus*. Adv Parasitol.

[21] Torgerson PR, Keller K, Magnotta M, Ragland N (2010). The global burden of alveolar echinococcosis. PLoS Negl Trop Dis.

[22] Budke CM, Deplazes P, Torgerson PR (2006). Global socioeconomic impact of cystic echinococcosis. Emerg Infect Dis.

[23] Vuitton DA, McManus DP, Rogan MT, Romig T, Gottstein B, Naidich A (2020). International consensus on terminology to be used in the field of echinococcoses. Parasite.

[24] Wu W, Wang H, Wang Q, Zhou X, Wang L, Zheng C (2018). A nationwide sampling survey on echinococcosis in China during 2012–2016. Zhongguo Ji Sheng Chong Xue Yu Ji Sheng Chong Bing Za Zhi.

[25] Hu D, Song X, Wang N, Zhong X, Wang J, Liu T (2015). Molecular identification of *Echinococcus granulosus* on the Tibetan Plateau using mitochondrial DNA markers. Genet Mol Res.

[26] Yan N, Nie HM, Jiang ZR, Yang AG, Deng SJ, Guo L (2013). Genetic variability of *Echinococcus granulosus* from the Tibetan plateau inferred by mitochondrial DNA sequences. Vet Parasitol.

[27] Ohiolei JA, Xia CY, Li L, Liu JZ, Tang WQ, Wu YT (2019). Genetic variation of *Echinococcus* spp. in yaks and sheep in the Tibet Autonomous Region of China based on mitochondrial DNA. Parasites Vectors.

[28] Li B, Quzhen G, Xue CZ, Han S, Chen WQ, Yan XL (2019). Epidemiological survey of echinococcosis in Tibet Autonomous Region of China. Infect Dis Poverty.

[29] WHO Informal Working Group on Echinococcosis (1996). Guidelines for treatment of cystic and alveolar echinococcosis in humans. WHO informal working group on Echinococcosis. Bull World Health Organ.

[30] Brunetti E, Kern P, Vuitton DA (2010). Expert consensus for the diagnosis and treatment of cystic and alveolar echinococcosis in humans. Acta Trop.

[31] Boufana B, Umhang G, Qiu J, Chen X, Lahmar S, Boue F (2013). Development of three PCR assays for the differentiation between *Echinococcus shiquicus*, *E. granulosus* (G1 genotype), and *E. multilocularis* DNA in the co-endemic region of Qinghai-Tibet plateau, China. Am J Trop Med Hyg.

[32] Sambrook J, Fritsch EF, Maniatis T (1989). Molecular cloning: a laboratory manual.

[33] Mak SST, Gopalakrishnan S, Carøe C, Geng C, Liu S, Sinding MS (2017). Comparative performance of the BGISEQ-500 vs Illumina HiSeq2500 sequencing platforms for palaeogenomic sequencing. GigaScience.

[34] Kinkar L, Laurimae T, Sharbatkhori M, Mirhendi H, Kia EB, Ponce-Gordo F (2017). New mitogenome and nuclear evidence on the phylogeny and taxonomy of the highly zoonotic tapeworm *Echinococcus granulosus* sensu stricto. Infect Genet Evol.

[35] Laurimae T, Kinkar L, Andresiuk V, Haag KL, Ponce-Gordo F, Acosta-Jamett G (2016). Genetic diversity and phylogeography of highly zoonotic *Echinococcus granulosus* genotype G1 in the Americas (Argentina, Brazil, Chile and Mexico) based on 8279bp of mtDNA. Infect Genet Evol.

[36] Kinkar L, Laurimae T, Simsek S, Balkaya I, Casulli A, Manfredi MT (2016). High-resolution phylogeography of zoonotic tapeworm *Echinococcus granulosus* sensu stricto genotype G1 with an emphasis on its distribution in Turkey, Italy and Spain. Parasitology.

[37] Dierckxsens N, Mardulyn P, Smits G (2017). NOVOPlasty: de novo assembly of organelle genomes from whole genome data. Nucleic Acids Res.

[38] Katoh K, Misawa K, Kuma K, Miyata T (2002). MAFFT: a novel method for rapid multiple sequence alignment based on fast Fourier transform. Nucleic Acids Res.

[39] Hall TA. BioEdit: a user-friendly biological sequence alignment editor and analysis program for Windows 95/98/NT. In: Nucleic Acids Symp Ser. 1999; London: Information Retrieval Ltd., c1979-c2000. p. 95–8.

[40] Kalyaanamoorthy S, Minh BQ, Wong TKF, von Haeseler A, Jermiin LS (2017). ModelFinder: fast model selection for accurate phylogenetic estimates. Nat Methods.

[41] Nguyen LT, Schmidt HA, von Haeseler A, Minh BQ (2015). IQ-TREE: a fast and effective stochastic algorithm for estimating maximum-likelihood phylogenies. Mol Biol Evol.

[42] Minh BQ, Nguyen MA, von Haeseler A (2013). Ultrafast approximation for phylogenetic bootstrap. Mol Biol Evol.

[43] Guindon S, Dufayard JF, Lefort V, Anisimova M, Hordijk W, Gascuel O (2010). New algorithms and methods to estimate maximum-likelihood phylogenies: assessing the performance of PhyML 3.0. Syst Biol.

[44] Knapp J, Nakao M, Yanagida T, Okamoto M, Saarma U, Lavikainen A (2011). Phylogenetic relationships within *Echinococcus* and Taenia tapeworms (Cestoda: Taeniidae): an inference from nuclear protein-coding genes. Mol Phylogenet Evol.

[45] Leigh JW, Bryant D (2015). POPART: full-feature software for haplotype network construction. Methods Ecol Evol.

[46] Tajima F (1989). Statistical method for testing the neutral mutation hypothesis by DNA polymorphism. Genetics.

[47] Excoffier L, Laval G, Schneider S (2007). Arlequin (version 3.0): an integrated software package for population genetics data analysis. Evol Bioinform Online.

[48] Fu YX (1997). Statistical tests of neutrality of mutations against population growth, hitchhiking and background selection. Genetics.

[49] Rozas J, Ferrer-Mata A, Sanchez-DelBarrio JC, Guirao-Rico S, Librado P, Ramos-Onsins SE (2017). DnaSP 6: DNA sequence polymorphism analysis of large data sets. Mol Biol Evol.

[50] Lemey P, Rambaut A, Drummond AJ, Suchard MA (2009). Bayesian phylogeography finds its roots. PLoS Comput Biol.

[51] Suchard MA, Lemey P, Baele G, Ayres DL, Drummond AJ, Rambaut A (2018). Bayesian phylogenetic and phylodynamic data integration using BEAST 1.10. Virus Evol.

[52] Ayres DL, Darling A, Zwickl DJ, Beerli P, Holder MT, Lewis PO (2012). BEAGLE: an application programming interface and high-performance computing library for statistical phylogenetics. Syst Biol.

[53] Rambaut A, Drummond AJ, Xie D, Baele G, Suchard MA (2018). Posterior summarization in Bayesian phylogenetics using Tracer 1.7. Syst Biol.

[54] Bielejec F, Baele G, Vrancken B, Suchard MA, Rambaut A, Lemey P (2016). Sprea D3: interactive visualization of spatiotemporal history and trait evolutionary processes. Mol Biol Evol.

[55] Kawakubo S, Gao F, Li S, Tan Z, Huang YK, Adkar-Purushothama CR (2021). Genomic analysis of the brassica pathogen turnip mosaic potyvirus reveals its spread along the former trade routes of the Silk Road. Proc Natl Acad Sci USA.

[56] WHO Informal Working Group (2003). International classification of ultrasound images in cystic echinococcosis for application in clinical and field epidemiological settings. Acta Trop.

[57] Nakao M, Yanagida T, Konyaev S, Lavikainen A, Odnokurtsev VA, Zaikov VA (2013). Mitochondrial phylogeny of the genus *Echinococcus* (Cestoda: Taeniidae) with emphasis on relationships among *Echinococcus canadensis* genotypes. Parasitology.

[58] Wang N, Xie Y, Liu T, Zhong X, Wang J, Hu D (2016). The complete mitochondrial genome of G3 genotype of *Echinococcus granulosus* (Cestoda: Taeniidae). Mitochondrial DNA A DNA Mapp Seq Anal.

[59] Wang Q, Yang L, Wang Y, Zhang GJ, Zhong B, Wu WP (2020). Disease burden of echinococcosis in Tibetan communities—a significant public health issue in an underdeveloped region of western China. Acta Trop.

[60] Lv FH, Peng WF, Yang J, Zhao YX, Li WR, Liu MJ (2015). Mitogenomic meta-analysis identifies two phases of migration in the history of eastern Eurasian sheep. Mol Biol Evol.

[61] Nakao M, Yokoyama N, Sako Y, Fukunaga M, Ito A (2002). The complete mitochondrial DNA sequence of the cestode *Echinococcus multilocularis* (Cyclophyllidea: Taeniidae). Mitochondrion.

[62] Zhao Y, Shen S, Jin X, Wang W, Li J, Chen W (2021). Cell-free DNA as a diagnostic tool for human echinococcosis. Trends Parasitol.

[63] Yang YR, McManus DP, Huang Y, Heath DD (2009). *Echinococcus granulosus* infection and options for control of cystic echinococcosis in Tibetan communities of Western Sichuan Province, China. PLoS Negl Trop Dis.

[64] Kinkar L, Laurimae T, Acosta-Jamett G, Andresiuk V, Balkaya I, Casulli A (2018). Distinguishing *Echinococcus granulosus* sensu stricto genotypes G1 and G3 with confidence: a practical guide. Infect Genet Evol.

[65] Zhu GQ, Yan HB, Li L, Ohiolei JA, Wu YT, Li WH (2020). First report on the phylogenetic relationship, genetic variation of *Echinococcus shiquicus* isolates in Tibet Autonomous Region, China. Parasites Vectors.

[66] Shang J, Zhang G, Yu W, He W, Wang Q, Zhong B (2019). Molecular characterization of human echinococcosis in Sichuan, Western China. Acta Trop.

[67] Heath DD, Zhang LH, McManus DP (2005). Short report: inadequacy of yaks as hosts for the sheep dog strain of *Echinococcus granulosus* or for *E. Multilocularis*. Am J Trop Med Hyg.

[68] Joshi DD, Joshi AB, Joshi H (1997). Epidemiology of echinococcosis in Nepal, Southeast Asian. J Trop Med Public Health.

[69] Kinkar L, Korhonen PK, Cai H, Gauci CG, Lightowlers MW, Saarma U (2019). Long-read sequencing reveals a 4.4 kb tandem repeat region in the mitogenome of *Echinococcus granulosus* (sensu stricto) genotype G1. Parasites Vectors.

[70] Sadjjadi SM, Mikaeili F, Karamian M, Maraghi S, Sadjjadi FS, Shariat-Torbaghan S (2013). Evidence that the *Echinococcus granulosus* G6 genotype has an affinity for the brain in humans. Int J Parasitol.

