# Supplementary figures and images for: Echinococcus spp. and genotypes infecting humans in Tibet Autonomous Region of China: a molecular investigation with near-complete/complete mitochondrial sequences
Source: Parasit Vectors. 2022 Mar 5;15:75. doi: 10.1186/s13071-022-05199-6 (PMC8898537; doi:10.1186/s13071-022-05199-6)

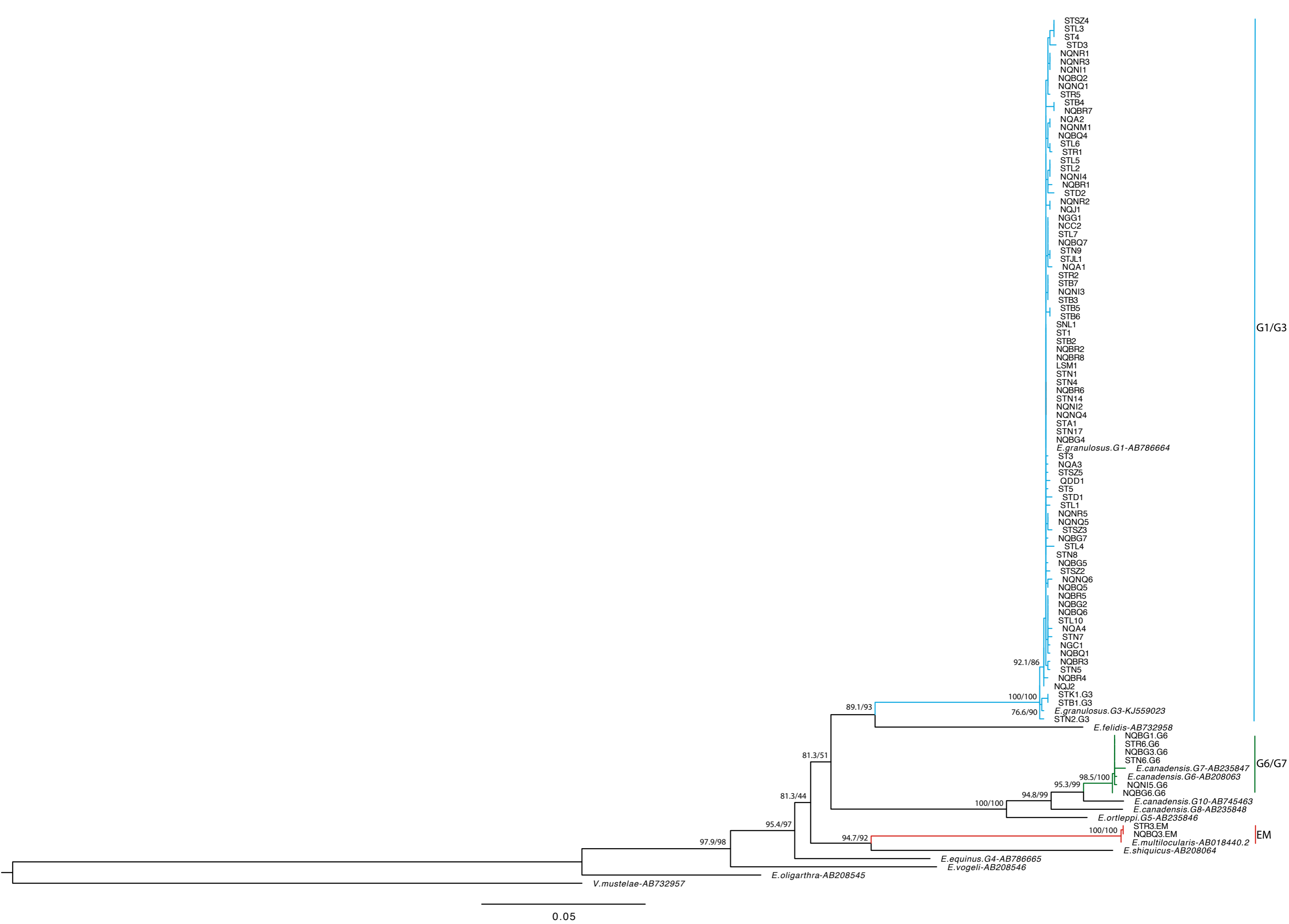

Supplement: Supplementary file 1 — Additional file 1: Figure S1. Phylogenetic tree of Echinococcus spp. and genotypes with a maximum-likelihood approach using the cox1 gene sequences (92 from this study and 14 references). [file 13071_2022_5199_MOESM1_ESM.pdf]

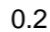

Supplement: Supplementary file 2 — Additional file 2: Figure S2. Phylogenetic tree of Echinococcus spp. and genotypes with a maximum-likelihood approach using the nad1 gene sequences (92 from this study and 14 references). [file 13071_2022_5199_MOESM2_ESM.pdf]

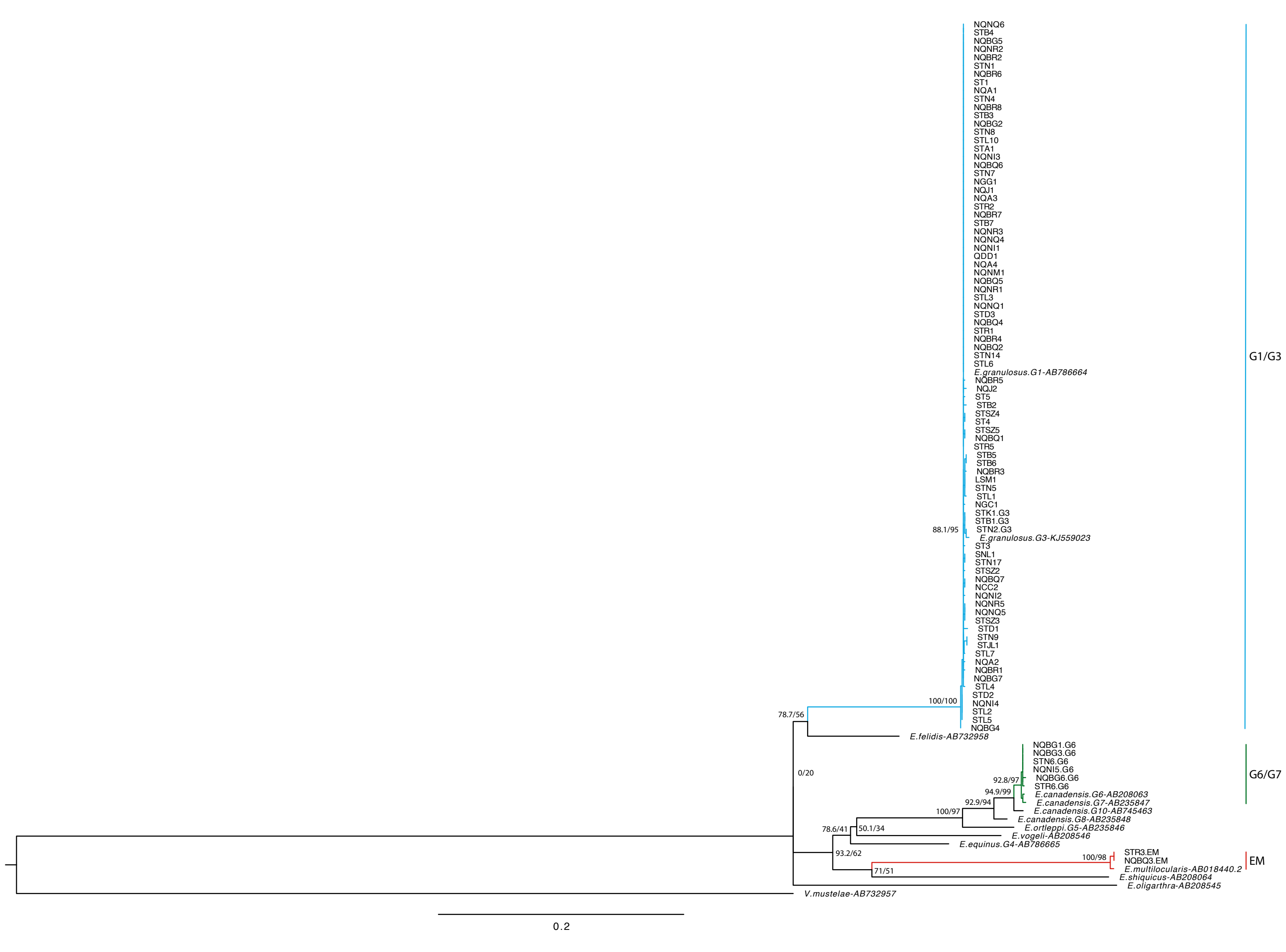

Supplement: Supplementary file 3 — Additional file 3: Figure S3. Phylogenetic tree of Echinococcus spp. and genotypes with a maximum-likelihood approach using the nad2 gene sequences (92 from this study and 14 references). [file 13071_2022_5199_MOESM3_ESM.pdf]

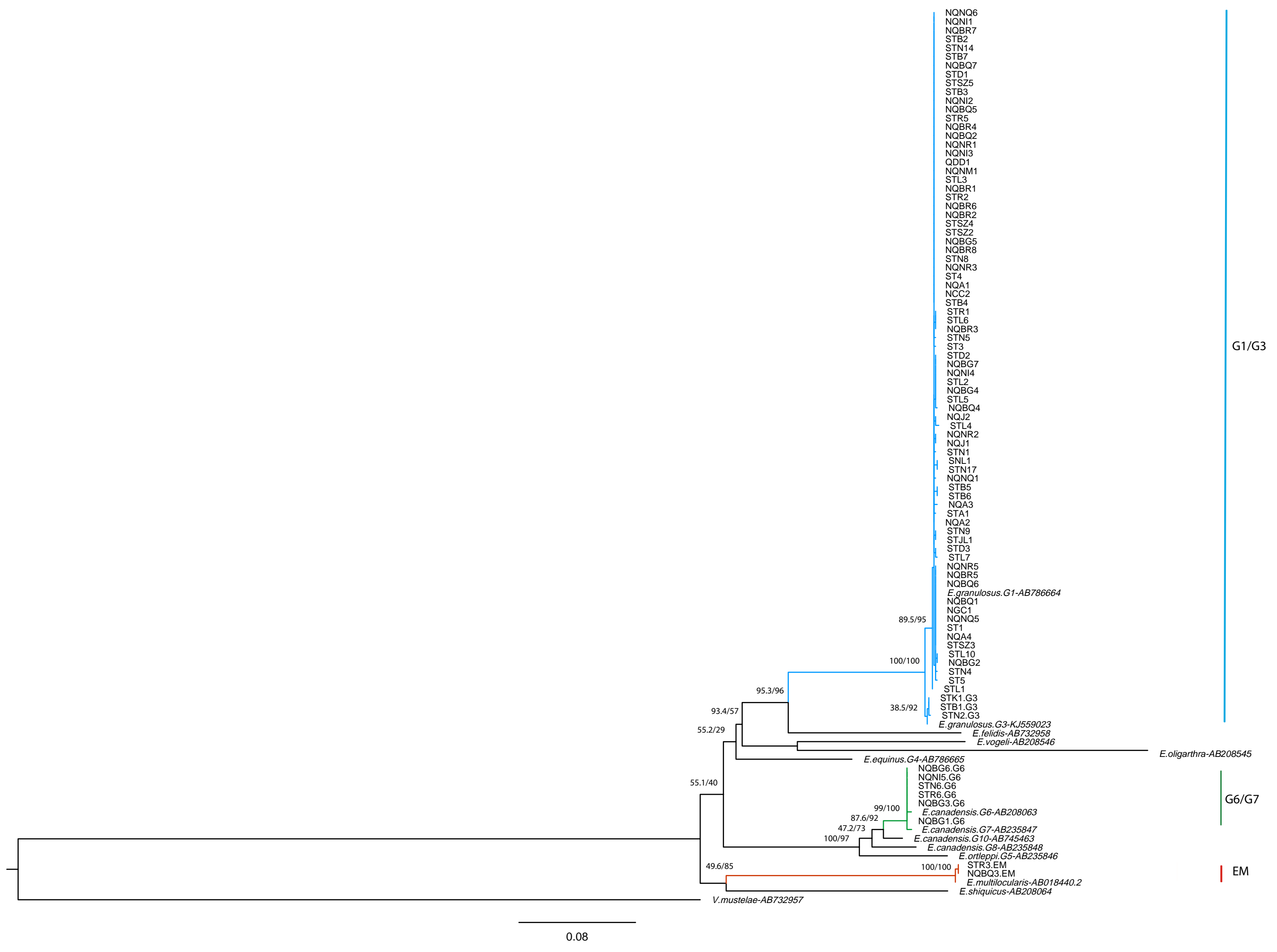

Supplement: Supplementary file 4 — Additional file 4: Figure S4. Phylogenetic tree of Echinococcus spp. and genotypes with a maximum-likelihood approach using the nad5 gene sequences (88 from this study and 14 references). [file 13071_2022_5199_MOESM4_ESM.pdf]

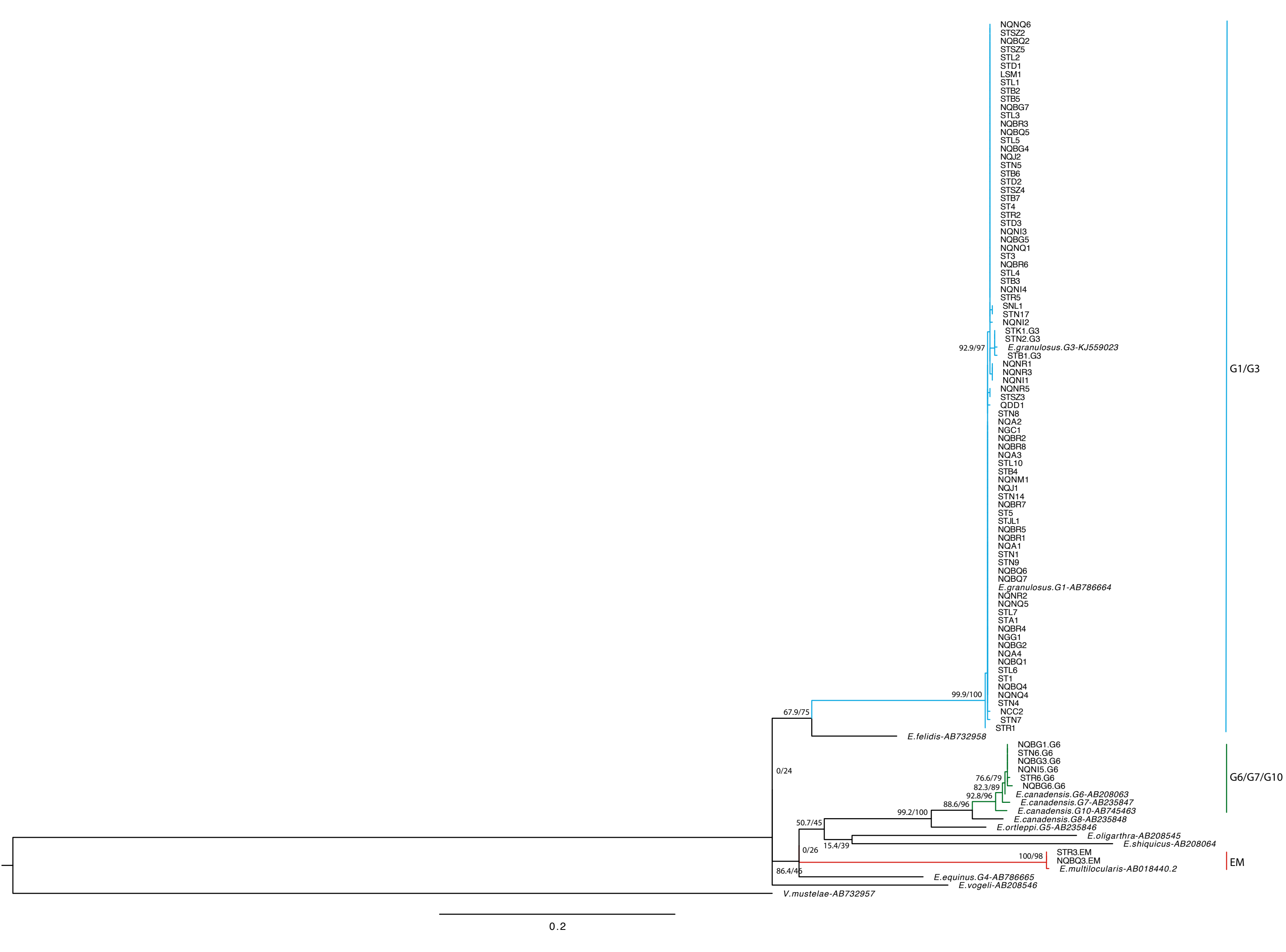

Supplement: Supplementary file 5 — Additional file 5: Figure S5. Phylogenetic tree of Echinococcus spp. and genotypes with a maximum-likelihood approach using the atp6 gene sequences (92 from this study and 14 references). [file 13071_2022_5199_MOESM5_ESM.pdf]
