# Supplementary material for: Echinococcus spp. and genotypes infecting humans in Tibet Autonomous Region of China: a molecular investigation with near-complete/complete mitochondrial sequences
Source: Parasit Vectors. 2022 Mar 5;15:75. doi: 10.1186/s13071-022-05199-6 (PMC8898537; doi:10.1186/s13071-022-05199-6)
Supplement: Supplementary file 6 — Additional file 6: Table S1. Detailed information of the patients and the Echinococcus spp. and genotypes identified (obtained from the maximum-likelihood method). [file 13071_2022_5199_MOESM6_ESM.pdf]

Code	City	County	Age	Sex	Sample type	Cyst size (mm)	Average Cyst size (mm)	Viable protoscoleces	Classification	NGS/PCR+NGS	qPCR	Genotype/species	No of cysts	Number of cyst-removal surgery
NQBG7	Naqu	Bange	56	Female	Endocyst	L-36*66	51	··	CE4	PCR+NGS	*Eg*[a]	G1	1	1
NQBR6	Naqu	Biru	29	Female	Endocyst	L-40*56	48	··	CE4	PCR+NGS	*Eg*	G1	1	1
ST3	Shigatse	NA	37	Male	Endocyst	R-94*100	97	Yes	CE2	PCR+NGS	*Eg*	G1	1	··
NQBG4	Naqu	Bange	46	Male	Endocyst	R-82*63	73	No	CE3	PCR+NGS	*Eg*	G1	1	1
STD2	Shigatse	Dingri	32	Female	Endocyst	R-48*48	48	··	CE3	PCR+NGS	*Eg*	G1	1	1
STL5	Shigatse	Lazi	13	Female	Endocyst	R-35*35	35	Yes	CE1	NGS	*Eg*	G1	1	1
STL2	Shigatse	Lazi	35	Male	Endocyst	R-110*139	125	No	CE1	NGS	*Eg*	G1	1	1
NQNI4	Naqu	Nima	48	Male	Cyst fluid	R-77*76	77	Yes	CE2/CE3	NGS	*Eg*	G1	1	1
STL4	Shigatse	Lazi	33	Male	Endocyst	R-68*77	73	No	CE2	NGS	*Eg*	G1	1	··
STSZ2	Shigatse	Sangzhuzi	50	Female	Endocyst	R-100*93	97	No	CE1	NGS	*Eg*	G1	1	1
NQJ2	Naqu	Jiali	61	Female	Endocyst	R-102*103	103	No	CE2/CE4	PCR+NGS	*Eg*	G1	1	··
STSZ5	Shigatse	Sangzhuzi	42	Male	Endocyst	R-89*67	78	Yes	CE3	PCR+NGS	*Eg*	G1	1	··
STD1	Shigatse	Dingri	52	Female	Endocyst	R-125*125	125	··	CE2	PCR+NGS	*Eg*	G1	1	1
NQBQ5	Naqu	Baqing	28	Female	Endocyst	R-140*112	126	··	CE2/CE4	NGS	*Eg*	G1	1	1
STB2	Shigatse	Bailang	17	Female	Endocyst	R-43*40 & R-75*60	68	No	CE1/CE3	NGS	*Eg*	G1	2	1
NQBG5	Naqu	Bange	31	Female	Endocyst	L-106*73	90	··	CE2	PCR+NGS	*Eg*	G1	1	1
STL1	Shigatse	Lazi	12	Female	Endocyst	R-91*76	84	No	CE1	NGS	*Eg*	G1	1	1
STB5	Shigatse	Bailang	45	Female	Cyst fluid	L-60*60&50*56	60	Yes	CE4	NGS	··	G1	2	1
STB6	Shigatse	Bailang	59	Female	Endocyst	R-80*84	82	No	CE2	NGS	··	G1	>3	··
LSM1	Lhasa	Mozhu Gongka	20	Female	Endocyst	R-81*75	78	No	CE3	PCR+NGS	*Eg*	G1	1	··
NQBR3	Naqu	Biru	34	Male	Endocyst	R-176*175	176	No	CE2	NGS	*Eg*	G1	1	1
STN5	Shigatse	Nanmulin	11	Male	Endocyst	R-47*44	46	Yes	CE1	NGS	Failed	G1	1	1
NQNI1	Naqu	Nima	43	Male	Endocyst	R-97*91	94	··	CE2	NGS	*Eg*	G1	1	1
NQNR1	Naqu	Nierong	18	Female	Endocyst	R-100*90 & Pelvic 44*45	95	No	Liver-CE1/Pelvic-CE2	NGS	*Eg*	G1	2	1
NQNR3	Naqu	Nierong	35	Male	Endocyst	R-89*69	79	··	CE4	PCR+NGS	*Eg*	G1	1	1
NQNQ6	Naqu	Naqu	36	Female	Endocyst	R-110*119&R-55*69	115	Yes	CE1/CE3	PCR+NGS	*Eg*	G1	2	··
NQBQ2	Naqu	Baqing	44	Female	Endocyst	Liver-49*58 & 60*63 & 66*50	62	··	CE4	NGS	*Eg*	G1	3	1
STD3	Shigatse	Dingri	30	Female	Endocyst	R-76*79	78	No	CE2	PCR+NGS	*Eg*	G1	1	··
NQNQ1	Naqu	Naqu	24	Male	Endocyst	L-75*73	49	··	CE1	NGS	*Eg*	G1	1	1
STR5	Shigatse	Renbu	23	Male	Endocyst	··	··	No	··	PCR+NGS	*Eg*	G1	··	··
STL3	Shigatse	Lazi	23	Female	Endocyst	L-125*67	96	No	CE1	NGS	*Eg*	G1	1	1
STSZ4	Shigatse	Sangzhuzi	15	Male	Endocyst	R-114*93	104	Yes	CE1	PCR+NGS	*Eg*	G1	1	··
ST4	Shigatse	NA	40	Female	Endocyst	R-99*124 &Pelvic-62*45&27*30	112	··	R-CE2/CE4	PCR+NGS	*Eg*	G1	3	··
SNL1	Shannan	Langkazi	55	Male	Endocyst	R-164*110 &L-14*16	137	No	R-CE2/L-CE3	PCR+NGS	*Eg*	G1	2	··

STN17	Shigatse	Nanmulin	32	Female	Endocyst	R-103*90	97	Yes	CE3	PCR+NGS	*Eg*	G1	1	··
NQNI2	Naqu	Nima	47	Male	Endocyst	R-86*94&L-23*18	90	··	R-CE4/L-CE5	PCR+NGS	*Eg*	G1	2	1
STB3	Shigatse	Bailang	34	Female	Endocyst	R-86*67	77	··	CE2	PCR+NGS	*Eg*	G1	1	1
STB7	Shigatse	Bailang	47	Female	Endocyst	R-78*81	80	··	CE4	PCR+NGS	*Eg*	G1	1	1
NQNI3	Naqu	Nima	23	Female	Endocyst	R-45*48	47	No	CE3	PCR+NGS	*Eg*	G1	1	1
STR2	Shigatse	Renbu	5	Male	Endocyst	R-36*32 & L-34*32 & 23*18	34	No	CE3	NGS	*Eg*	G1	3	1
STR1	Shigatse	Renbu	14	Male	Endocyst	L-54*58 & 14*20	56	No	CE2	NGS	*Eg*	G1	2	··
STL6	Shigatse	Lazi	59	Female	Endocyst	L-114*102	108	··	CE2	PCR+NGS	*Eg*	G1	1	1
NQBQ4	Naqu	Baqing	45	Male	Endocyst	R-60*63	62	··	··	NGS	*Em*+G1	G1	1	3
NQA2	Naqu	Ando	39	Female	Endocyst	R-80*89 & Abdomen-73*53&57*41&43*41	85	No	Liver-CE1/Abdomen-CE1/CE2/CE4	NGS	*Eg*	G1	4	··
NQNM1	Naqu	Nama	36	Female	Endocyst	L-102*77	90	Yes	CE2	NGS	*Eg*	G1	1	1
NQBR1	Naqu	Biru	38	Female	Endocyst	R-135*187	161	No	CE1	NGS	*Eg*	G1	1	1
STN14	Shigatse	Nanmulin	56	Female	Endocyst	R-140*154	147	Yes	CE3	PCR+NGS	*Eg*	G1	1	··
NQA3	Naqu	Ando	28	Female	Endocyst	R-29*30 & R-33*30	32	··	CE5/CE4	PCR+NGS	*Eg*	G1	2	1
QDD1	Qamdo	Dingqing	50	Female	Endocyst	L-96*106	101	No	CE1	NGS	*Eg*	G1	1	1
STA1	Shigatse	Angren	10	Female	Endocyst	R-65*60	63	Yes	CE1	NGS	*Eg*	G1	1	1
NQNQ4	Naqu	Naqu	45	Female	Endocyst	R60*48	54	··	CE4	PCR+NGS	··	G1	1	1
NQBR8	Naqu	Biru	55	Male	Endocyst	R-81*60	71	··	CE4	PCR+NGS	*Eg*	G1	1	1
STN1	Shigatse	Nanmulin	14	Male	Endocyst	R-16*45&L-37*51&18*19	44	No	R-CE1/L-CE4	NGS	*Eg*	G1	3	1
NQBR2	Naqu	Biru	28	Male	Endocyst	R-104*91	98	No	CE1	NGS	*Eg*	G1	1	1
STN8	Shigatse	Nanmulin	21	Female	Endocyst	R-78*80	79	··	CE2	NGS	··	G1	1	··
STL7	Shigatse	Lazi	43	Female	Endocyst	Abdomen-62*36	49	··	CE4	PCR+NGS	*Eg*	G1	1	3
STN9	Shigatse	Nanmulin	38	Female	Endocyst	R-89*110	100	Yes	CE4	NGS	··	G1	1	1
STJL1	Shigatse	Jilong	24	Male	Endocyst	Spleen-87*93	90	No	CE4	PCR+NGS	*Eg*	G1	1	1
NGG1	Ngari	Gaize	21	Male	Endocyst	Liver-135*89	112	··	CE2/CE4	PCR+NGS	*Eg*	G1	1	··
NQBQ7	Naqu	Baqing	29	Male	Endocyst	R-122*92	107	No	CE2	PCR+NGS	*Eg*	G1	1	··
NCC2	Nyingchi	Chayu	19	Female	Endocyst	R-70*77 & Spleen 47*49	74	No	R-CE4	PCR+NGS	*Eg*	G1	2	··
NQA1	Naqu	Ando	61	Female	Endocyst	R-133*86 & 113*84	110	Yes	CE1	NGS	*Eg*	G1	2	1
NQNR2	Naqu	Nierong	13	Female	Endocyst	L, R-111*95 & Spleen-91*58	103	No	CE1	NGS	*Eg*	G1	2	1
NQJ1	Naqu	Jiali	25	Female	Endocyst	L, R-109*74	92	··	CE4	NGS	Failed	G1	>3	1
NQBR4	Naqu	Biru	24	Female	Endocyst	R-111*173	142	No	CE2	NGS	*Eg*	G1	1	1
STB4	Shigatse	Bailang	5	Female	Cyst fluid	R-141*154	148	Yes	CE4	NGS	··	G1	1	··
NQBR7	Naqu	Biru	27	Male	Cyst fluid	L-88*75 & R-25*27	82	No	CE2/CE5	NGS	··	G1	2	1
ST5	Shigatse	NA	12	Female	Endocyst	R-55*62	59	No	CE2	PCR+NGS	*Eg*	G1	1	1
ST1	Shigatse	NA	8	Female	Endocyst	R-45*58	52	No	CE4	PCR+NGS	*Eg*	G1	1	··

STN4	Shigatse	Nanmulin	22	Female	Endocyst	R-49*44	47	··	CE4	NGS	*Eg*	G1	1	1
NQNQ5	Naqu	Naqu	54	Female	Endocyst	R-106*96	101	No	CE4	PCR+NGS	*Eg*	G1	1	··
NQNR5	Naqu	Nierong	15	Female	Endocyst	R-81*66	74	Yes	CE2	PCR+NGS	*Eg*	G1	1	··
STSZ3	Shigatse	Sangzhuzi	10	Female	Endocyst	L-55*66	61	No	CE1	NGS	*Eg*	G1	2	1
NGC1	Ngari	Cuole	19	Female	Endocyst	L-80*58	69	No	CE3	PCR+NGS	*Eg*	G1	1	1
STL10	Shigatse	Lazi	28	Male	Endocyst	R-133*160	147	No	CE2/CE4	PCR+NGS	*Eg*	G1	1	1
NQBG2	Naqu	Bange	15	Female	Endocyst	R-130*100	115	No	CE1	NGS	*Eg*	G1	1	2
NQBQ1	Naqu	Baqing	13	Female	Endocyst	R-46*62 & 15*17	54	··	CE3/CE4/CE5	NGS	*Eg*	G1	2	1
NQBR5	Naqu	Biru	28	Female	Endocyst	R-46*41	44	Yes	CE1	NGS	*Eg*	G1	1	··
NQBQ6	Naqu	Baqing	32	Female	Endocyst	Pelvic-57*50	54	No	··	NGS	Failed	G1	1	1
NQA4	Naqu	Ando	33	Female	Endocyst	R-80*80	80	··	CE3	PCR+NGS	*Eg*	G1	1	1
STR3	Shigatse	Renbu	34	Male	Endocyst	R-145*121	133	··	··	NGS	*Em*[b]	*Em*	1	1
NQBQ3	Naqu	Baqing	37	Female	Endocyst	R-56*56	56	··	AE	NGS	*Em*+G1	*Em*	1	1
STK1	Shigatse	Kangma	29	Female	Endocyst	R-129*81	105	No	CE1	NGS	*Eg*	G3	1	··
STB1	Shigatse	Bailang	28	Female	Endocyst	R-157*159	158	No	CE1	NGS	*Eg*	G3	1	1
STN2	Shigatse	Nanmulin	54	Female	Endocyst	R-76*70	73	No	CE1	NGS	*Eg*	G3	1	1
NQBG6	Naqu	Bange	28	Female	Endocyst	R-57*57	57	No	CE3	PCR+NGS	*Eg*	G6	1	1
STN6	Shigatse	Nanmulin	30	Male	Cyst fluid	R-118*98 & L-65*60	108	Yes	CE3	NGS	*Eg*	G6	2	··
NQBG3	Naqu	Bange	30	Female	Endocyst	R-56*42	49	··	CE3	PCR+NGS	*Eg*	G6	1	1
NQBG1	Naqu	Bange	29	Female	Endocyst	R-78*63	71	No	CE3	NGS	*Eg*	G6	1	1
STR6	Shigatse	Renbu	15	Male	Endocyst	L-43*52	48	··	CE1	PCR+NGS	*Eg*	G6	1	1
NQNI5	Naqu	Nima	16	Female	Endocyst	R-49*38	44	No	CE3	NGS	*Eg*	G6	1	1
STN7	Shigatse	Nanmulin	10	Male	Endocyst	R-44*43	44	No	CE1	NGS	*Eg*	G1	1	1

NA: not applicable

Eg[a]: *E. granulosus* (G1 genotype)

Em[b]: *E. multilocularis*

L: left liver; R: right liver
